# The Wonders of Modern Medicine and Surgery

**Published:** 1897-12

**Authors:** Lewis H. Watson

**Affiliations:** Chicago, Ill.


### THE WONDERS OF MODERN MEDICINE AND SURGERY.

By LEWIS H. WATSON, M. D., CHICAGO, ILL.

It is greatly to the detriment of physicians and surgeons the world over, that so little effort is made by them to interest or inform the general public, in a popular way, in regard to the great work upon which they are unceasingly engaged. This work is twofold; the alleviation of human suffering, and the relentless warfare they are making upon every form of disease which threatens and imperils human existence. Occasionally some brief notice meets the eye, in the daily papers, of a wonderfully skillful operation performed, or the discovery of some new germ by which to diagnosticate disease, and repel its ravages. But the mention is speedily forgotten. Only in the pages of their medical journals is the matter kept alive, and its merits reviewed; and then, in such a technical way, that to laymen it remains a sealed book. Oftener than otherwise, the reference in the daily press is satirical or humorous, allusion is made to bugs, microbes, microbe-killers, pill-peddlers, quacks, and charlatans. The true physician shrinks from writing for the public about his work. It may bring upon him the imputation of being an advertiser, or, he may fancy that as the practice of medicine is a great mystery to the public, he will be more highly considered if it remains so. This feeling is taken advantage of by the real quack, and at the first rumor of a new germ discovered, or a new appliance to be used in surgery, he seizes upon in it with avidity, searches the medical periodicals for an account of it, publishes a cut in the papers, and buys the instrument at whatever cost, to exhibit in his reception-room.

Conscious of the dignity and grandeur of his profession, and secure in the knowledge that when in real trouble the dear general public will certainly seek him out, the earnest physician and surgeon smiles, and quietly resumes his labors. The world does not understand or appreciate his work until he is needed. What that work is, the skill, patience, learning, and at times even genius, which has adorned and glorified it, through man's courage, ambition and masterly intuition is the object of this article.

No better definition of life has ever been given than that of Bichat, who died at thirty-three a martyr to the cause of human affliction. "Life is the sum of the forces by which death is resisted." To make this resistance a stable one, to unite the forces of the human organism so completely as to justify the assertion of the late Sir Benjamin Ward Richardson, that "man should live not to three score years and ten, but to an even one hundred years," is the cause for which the profession works. The enormous strides made by medicine and surgery in the past quarter of a century eclipse the feats performed in this line during any other epoch the student of medical and surgical literature may select. They rival indeed the discoveries made in any other line of scientific work, and surpass *all* lines in the one great feature which measures the achievements of man's intellect—the prolongation of human life. Only through the the most arduous and exhausting labors, the most painstaking and scientific experiments, the most exacting, and oftentimes dangerous and self-sacrificing devotion to the cause he has chosen, has the scientific physician and surgeon of the day become the magician he is; the alleviator of human suffering and the savior of the whole human race. To turn aside the spear of Azrael and renew life's waning fires is his mission and his hope.

How he has pursued this work, and how he has built up, step by step, the present system of medicine and surgery, until the percentage table will bear out his claim to scientific accuracy, I shall try to unfold. With the exception of the introduction of anæsthesia, his best work has been done in the past fifteen years.

Just fifty years ago, in the amphitheatre of the Massachusetts General Hospital, a young man was laid upon the operating table, and while under the influence of ether, administered by Dr. Morton, Dr. John C. Warren removed a tumor from his neck without pain or consciousness. Nothing so wonderful as this had been known in the world of medicine and surgery since Homer sang of Æsculapius's art, and great Jupiter—

whose sign we daily use—resenting the god of Medicine's interference in bringing back Hippolytus to life, destroyed him with a bolt. What pæans of praise should we poor mortals not have sung to think that pain was banished from our lives when under the surgeon's knife? What honors should not have been conferred upon Morton, Wells and Jackson, and yet—and yet—these men all died in obscurity and want. From that day to this, the misery and anguish which had heretofore racked the human frame was no longer known. The muscular strength of man and the delicate, shrinking tissues of woman, were never more to be called upon to endure the ordeal of the knife. With the sufferer deprived of consciousness the work was half accomplished. We have the recorded statements of the surgeons of those days, of the mental strain and dread with which they approached their self-imposed task. A well-known physician, who suffered the amputation of a leg, just before the reign of anæsthesia, has given a touching picture of the horror and despair which seized him when his brother physicians gathered around the operating table, upon which he lay bound; and the excruciating agony he felt when that cold steel was passed through his thigh. I have seen the most delicate of women, with the highest emotional temperament, go smiling to the operating room, from which she emerged with a new lease of life, uttering a benediction upon the discoverers of anæsthesia. The little child, the aged man, lie down and sleep until the surgeon calls them back to life and health. All this hath anæsthesia done. Marvelous indeed as a beneficent gift bestowed by man's genius upon man.

This termination of the nineteenth century can well be called the Golden Age of medicine and surgery. Twenty-five years ago, or little more than that, Sir Joseph Lister's—then Mr. Joseph Lister—attention was attracted to the fact that in fracture of the thigh, when the skin was unbroken by the sharp point of the bone protruding, union was rapid and without what is called surgical fever. At first he supposed, as he had been taught, that laceration of the soft tissues was the cause of the great danger and delay in healing, which accompanied compound fracture (fracture where the bone protruded). But doubt arose in his mind, and he decided that the simple fact of the broken skin could not account for the increased mortality in such cases. About this time the scientific world was stirred by the experiments of Louis Pasteur upon fermentation and putrefaction, and by the controversy between Pasteur, Pouchet and Dr. C. Bastian upon spontaneous generation. The former asserting that all life must proceed from life, whereas Pouchet and Bastian, declared that life was constantly originating "*de novo*" from the juxtaposition of certain organic elements favoring its development. The controversy was exceedingly interesting. Aristotle has declared that "all dry bodies which become damp, and all damp bodies which are dried, engender animal life." "Bees," according to Virgil, "are produced from the corrupted entrails of a young bull." Van Helmont was more humorous still. To produce a pot of mice, his receipt was this:

"It suffices to press a soiled shirt into the orifice of a vessel containing a little corn. After about twenty-one days the ferment from the soiled shirt, modified by the odor of the corn, effected the transmutation into mice. It is only necessary to pair them to prolong the species." An Italian named Redi, was the first to subject this question of spontaneous generation to a more attentive examination. He showed that maggots in meat, are not spontaneously generated, but that they are the larvæ of flies' eggs. It is only necessary to surround the meat with gauze before exposing it to the air. As no flies can come in contact with it, there are no maggots. It remained for Pasteur to demonstrate to the scientific world that spontaneous generation was a chimera. He heated flasks containing putrescible fluids to the boiling point, and then drew out the neck under the heat of a blow pipe, and sealed the flasks. While thus sealed, every germ having been killed by the boiling, and a vacuum existing between the mouth of the flask and the liquid within, no life became visible. It was only necessary to break the neck of part of the flasks and admit the air, to have the fluid swarming in a short time with germs—bacteria as they began to be called. The tale of antisepsis—originating with Sir Joseph Lister—can not be told without referring to these experiments of Pasteur. Having demolished the theory of spontaneous generation, Pasteur took up the study of fermentation and putrefaction. Schwann, in Germany, and Cagniard Latour, of France, about the same time discovered that there was life and movement in the cells of the yeast plant; and that the breaking up of sugar into alcohol, was somehow due to the micro-organisms contained in sweetwort ferments. Pasteur, convinced of the correctness of their views, began to study the yeast plant, and decided that the absence of change in wort coincided with the absence of foreign organisms. He made the assertion that all the diseases which wine and beer are subject to were the result of organic ferments, wafted to and fro in the air, or deposited upon the utensils used in the manufacture of wine and beer. This he proved conclusively both in France and England. By disease is meant the radical change which so affects the nature of these liquids, as to make them sour and unpalatable, and if kept any great length of time, absolutely putrid.

In writing about this Pasteur remarked: "There would be excellent opportunities for these germs introduced into the blood, to propagate, and become unwelcome guests, and even to endanger human life." And further: "Medical men agree that the question of contagion and infection will find their solution in the study of the ferments." Remember, that this was written by a man who was neither a physician nor a biologist, for Pasteur was educated as a chemist. The same idea seems to have penetrated the mind of Ambrose Pare, the great surgeon of the sixteenth century, who said, "A wound is not necessarily poisonous if kept from the air." Megatus, in the seventeenth century, stated that he believed there was something in the air prejudicial to the proper treatment of wounds. It remained for Sir Joseph Lister to develop these theories, and give his name to the modern treatment of wounds. In a personal letter written to Pasteur, in 1874, he says:

"Permit me to take the opportunity of offering you my hearty thanks for having demonstrated by your brilliant researches, the theory of putrefactive germs, and for having afforded me the means, the sole means, of perfecting the antiseptic system of surgery." Having learned from Pasteur that the putrefaction of animal tissues, and the fermentation in the must of wine, and the wort of beer, were caused by the action of certain micro-organisms, called ferments, he reasoned that if it were possible to keep these germs from coming in contact with open wounds, pus formation would be avoided. It had been noticed, that if the edges of wounds produced by a sharp cuttting instrument were brought in close adaptation, and maintained in this position, the wound healed without the formation of pus, or the supervention of fever. This action was at the time called "healing by first intention." When a wound was ragged or torn, the edges became everted, and after a few days, a thick, creamy fluid covered its surface. This was called "laudable pus," and was supposed to be necessary to the healing process. Lister conceived the idea that if he could find some substance strong enough to kill the micro-organisms without injuring the tissues, there would be no pus formation, which only caused sloughing, and retarded the return to a natural condition. By the advice of Dr. Anderson, professor of chemistry at Glasgow University, Lister tried carbolic acid, which was then nothing more than a mere chemical curiosity. He saturated gauze with a solution of the acid, and applied it to open wounds, covering the gauze with gold beaters' skin. He was delighted to find that his reasoning was correct, for the wounds healed without fever or the formation of pus. Here began antiseptic surgery. It was supposed that the germs floating in the air, were the cause of all this trouble, as they necessarily constantly fell upon the wounds. To avert this, and kill the germs, the air was sprayed with carbolic acid, and later with corrosive sublimate; the wound and the operator were also sprayed during an operation. It was some time after this, that it was learned that the germs of atmospheric air were not the fermented germs which caused putrefactions However, the value of Lister's discovery was incalculable. In Glasgow, where he worked, the mortality was 45 $\frac{7}{10}$ per cent. in all serious operations, mainly from septic poisoning. Soon after he introduced antiseptic methods, the mortality was reduced to 36 per cent. In comparison with other surgeons, his loss in the same series of cases was one to three.

For a long time, however, Lister's discovery was treated with scant notice by the medical journals, and derided by many of his colleagues, but others were led to adopt his methods, and Prof. Volkman stated in an address delivered about this time in London, that the last twelve cases of compound fracture of the femur he had treated, previous to the introduction of antisepsis, had all died. From that time, in 1881, until he delivered his address, he treated 135 cases by the antiseptic method without losing one. In the New York Hospital, Prof. Dennis states that, at that time, out of 126 cases of compound fracture, there were 61 deaths. Billroth, of Vienna, had a mortality of 40 per cent. before the introduction of antisepsis. The theory was now gaining ground over the entire world, with the result that the percentage of loss in serious cases was reduced to 15 per cent. Von Nussbaum, of the Algemeines Krankenhaus, in Munich, had great trouble with hospital gangrene, which was so prevalent that it was considered dangerous to perform the slightest operation. Under the Lister method, it entirely disappeared. The study of micro-organisms now became general; each one added his mite. Lister noticed in fracture of the ribs, where the lung was penetrated, and the pleural cavity opened, until the serum and blood had free access to the air, there was no decomposition. Tyndall found no germs after breathing into a ray of sunlight, and Gunning, of Amsterdam, showed that the expired air contained no germs capable of producing putrefactive fermentation. Strauss showed that sterilized beef bouillon could be breathed into half an hour, without developing bacteria therein. Miquel says, that although we inspire in tranquil air in a room, from 1,500 to 10,000 micro-organisms, the *expired air* is absolutely pure so far as germs are concerned. Very few get beyond the nasal passages; the moist mucus membrane, and the stiff hairs acting as traps to catch them. Among the experimenters were Lawson Tait and Koberle. These surgeons came to the conclusion that it was the coarser germs which attached themselves to the instruments used; the person of the patient or the operator, which set up the putrefactive fermentation, and the septic poisoning all feared so much. Experience proved this to be the case, and perfect cleanliness, not ordinary cleanliness, but surgical cleanliness—was found to produce such a wonderful lowering of the death-rate, that operations by the hundreds were performed without the occurrence of a single death from septic poisoning. What is meant by surgical cleanliness can only be explained by describing the preparations for a surgical operation by the modern surgeons of any large hospital in the world. The hands of the operating surgeon, assistants, and nurses, are thoroughly scrubbed with soap and water, using a nail brush, the surgeon even removing a ring if he wears one. The finger-nails are also very carefully cleaned. After this thorough cleansing the hands are dipped into alcohol, or have ether poured over them to remove all oily matter, which might harbor bacteria. The hands of the operator and assistants are now immersed in a solution of corrosive sublimate, or carbolic acid. The surgeon and his assistants wear long aprons, which have been boiled and freshly laundried. The person of the patient has already been just as carefully prepared, and is as surgically clean as that of the surgeons and assistants. Every instrument has been placed in boiling water, or subjected to the action of super-heated steam, and is now on a tray in an antiseptic bath. Every ligature has been boiled and preserved in a glass-stoppered bottle in an antiseptic. Every dressing and bit of gauze, and all sponges are equally as carefully prepared. Nothing can be more exquisitely clean than patient, surgeon, assistants and instruments. This is asepsis. This is the triumph of modern surgery, and to such a point of absolute perfection is the art brought at the present day, that except in a case of malignant disease, and gunshot wounds and injuries of a necessarily fatal character, death is the exception, and recovery the rule. All honor to Sir Joseph Lister, who lives to-day in a serene old age, honored by the medical and surgical profession, and the world at large, as one who by his foresight, has saved to humanity multitudes of valuable lives, and to friends and relatives their dearest and most beloved.

Medical science has had its victories also. Closely associated as it is with surgery, it is yet a science apart, for it deals more with hidden secrets. Surgery must necessarily be open It is a matter of technique and antisepsis. Medicine is more subtle, more involved and more obscure. The profound physician of the day must be chemist, philosopher, mind-reader and savant. The province of medicine is also wider. Besides disease, it deals with physiological chemistry, prophylaxis and hygiene. In hygiene alone, its banners are blazoned with victories. Victories so far-reaching and decisive, that the word plague or scourge, will in all likelihood never be written on them again. Witness the cholera epidemic which Hamburg experienced in 1892. Its business was prostrate, its population was decimated—the people dying by hundreds daily—its commerce was ruined, and its civic organization demoralized. Then a savior came, a modest physician, Dr. Robert Koch, of the Hygienic

Institute of Berlin. He looked over the field, and the pall of death was lifted. In a very ingenious and thorough manner, Dr. Koch, assisted by Dr. Dunbar, an American physician, and student of his, accomplished the perfect filtration of the water of the Elbe. Before he came it swarmed with vibrios, but he left it so pure that you may draw a cup from any hydrant and find it harmless. The streets are watered with it, it is used in the household, and it is potable without boiling. Gradually the dread scourge died out It returned slightly in '93. Tests made showed an alarming increase in cholera germs. Dr. Dunbar, who then, as now, had charge of the Hygienic Institute of Hamburg, ascertained that there was a bad leak in the tunnel which conveyed the water from the Kalte Hofe to the pumping works on the mainland. This was instantly stopped; and although surrounded by contaminated water, the Hamburg citizens drank the purified water of the same Elbe with impunity, and cholera disappeared. This superb hygienic supervision was carried to such an extent that the beer-mugs were scalded in boiling water and scoured. The cows even came under the scrutiny of Dr. Dunbar. Their udders were washed and disinfected before milking. All food products were examined critically. Was not this a triumph for medicine? Berlin, only 150 miles away, had a few sporadic cases of cholera. A few were imported to America, but the sanitary supervision at quarantine blocked its admission. It is now believed by the best authorities, that cholera can never gain a foothold in any civilized country as a scourge. Its reign of terror has passed. Who has done this? The physician.

I have spoken of Dr. Robert Koch; to this man—almost another Pasteur—we owe the discovery of the means of accurate diagnosis we now possess in cases of tuberculosis. In 1882 Koch exhibited samples of the stained bacillus tuberculosis before the Physiological Society of Berlin. His discovery has stood the test of time, and we know that every case of consumption must have this characteristic bacillus. Before 1882, it was a matter of opinion; physicians often differed. Now, if you find the bacillus, you have a case of phthisis. It is absolute. We not only learned that consumption was caused by a specific micro-organism, but we also learned that it was contagious, and although as yet we have not found the cure, we all look for it. Mortality has been lessened, cures have been, and are being made when known in time. Hygiene again here has taught us that we can limit its spread by destroying the sputum or matter expectorated by burning. If it be true, as asserted lately, that a specific microbe has been found in the blood, even before the bacilli are found in the sputum, we may reasonably hope that the day of the cure is not far distant. Analogy would suggest that if we can discover Laveran's "Plasmodium Malariæ," in the blood of malarial subjects

—for bacteriologists tell us that malaria is a parasitic disease, and its micro-organism one that destroys the red blood-corpuscle—if we can do this, why should we not expect that the blood of consumptives may also contain a microbe prejudicial to life? In fact, each of the fevers has its possible micro-organism, with certain peculiarities, changing not only the blood but the tissues. "These changes," says Meade Bolton, "may be temporary or permanent."

In 1883, the German government sent Dr. Koch to Egypt to investigate cholera, and this distinguished man soon found the cholera bacillus, the comma bacillus, as it is called from its peculiar shape, being curled on itself. Dr. Haffkine, a young Russian physician, has made pure cultures of this microbe, and declares that where tried in India, mortality has been lessened fifty per cent. by the use of the attenuated virus as an injection. It may happen that the gaunt spectre which for ages has terrorized Eastern nations, will, through this means, be rendered harmless. The inoculation of attenuated virus practiced by Dr. Haffkine is also a comparatively new discovery in medicine, and is designated as "Serum Therapy." Behring and Kitasato, of Berlin (students of Koch), were the leaders in this form of treatment. It is based upon the statement of these men that the blood of an animal rendered immune against a certain infectious disease, has the power, when injected into another living organism, of protecting the latter from a disease, or curing it. The bacillus of diphtheria, for instance, is introduced into a suitable culture medium, and an attenuated portion of this culture is injected into a horse as one of the healthiest of animals. This injection of a stronger degree of toxicity is repeated until an injection is used so strong, as Calmette says, that it is not exceeded in virulence by the most venomous of poisons. When this can be used without any reaction in the horse, his blood is called immune, and the serum of this blood is used to prepare the antitoxin we now use with so much success in diphtheria. The records of the six London hospitals showed in 1894 a loss of twenty-three per cent. in diphtheria cases. In 1895, 2,182 cases were treated with antitoxin, in the same hospital, with a death-rate of 7.5 per cent.; and Behring states he expects to reduce it to five per cent. It is scarcely fifteen years since the death-rate was fifty per cent. in all cases of epidemic diphtheria. The bacillus in tetanus (lockjaw), erysipelas, typhoid fever and influenza have been isolated and described; and the future serum therapy may be the greatest blow the constant and excessive use of drugs, indulged in by the American people, encouraged by their unrestrained advertising, may receive. One other subject, not exactly within the domain of medicine, except as medical science, covers a field in which is included all the studies made by scientists of conditions which affect the animal organism, and the means by which it resists the attacks made upon it, in the struggles for existence, I shall now refer to. It is what is called the doctrine of Phagocytosis, first suggested by our own Sternberg, and developed by Metschnikoff, of the Pasteur Institute. The human body, in fact, the bodies of all animals, are beset constantly by myriads of bacteria. They wage a constant warfare to obtain a foothold, and destroy organized matter; but within our bodies we also possess a defensive host, an army of cells called phagocytes. In the blood secretions and tissues these "infinitely little" cells keep up a constant, watchful promenade. Let but the skin be broken, or, through disease or some breach in the integrity of our defenses, an opportunity be offered for the besieging army of bacteria to effect an entrance, and this defensive garrison, ever on the alert, rush through blood vessels, tissues, and all obstructions, toward the part attacked. Each phagocyte seizes not one, but many of the invading bacteria, and swallows them, renders them inert, and then, crammed with the dangerous army, it makes its way outside the body and dies. Nothing could more fully illustrate the action of these phagocytes than what occurs when, during a walk, you jab your stick into an ant-hill. Then you see the scurrying horde fly to the point attacked, and try to repair the damage. So, in their unconscious, blind way, carrying out the theory of their organism, do the phagocytes, obedient to the instinct which governs their acts, unwittingly serve the host which entertains them; and thus is the integrity of our organism maintained.

It is perhaps not necessary to speak of the Roentgen rays. The discovery is too recent for us to know just what benefit, or in what direction the most benefit is to be derived. It is certain that the location of bullets, the diagnosis of dislocations from fractures, and the mapping out of the denser organs, like the heart, liver and kidneys, will certainly be accomplished. It is within the bounds of belief, that abscesses of liver, lungs and kidney, tumors and blood clots, the location of strictures, or foreign bodies, will undoubtedly be determined by the use of the wonderful rays.

We have taken but a cursory, and fleeting glance, at this Renaissance of Medicine and Surgery, which is now growing up. While far from complete in detail, it will, I hope, afford the lay reader a fair retrospect of what this branch of scientific research has done in the last quarter of a century for humanity. There is no greater leveler than sickness. There is no appeal made so strongly to any class or profession, as that made to the physician. He reads it in the eye of friend and patient. There is no incentive offered to man's sympathy and man's ambition like that which emanates from the sick-bed. The good physician cheers as the morning sunlight cheers, and he helps, as the good Samaritan helped. His compassion is boundless, and his courage is supreme. His motto is one from Ovid: "Res est Sacra Miser."—*N. E. Medical Monthly.*

